# IK Channel-Independent Effects of Clotrimazole and Senicapoc on Cancer Cells Viability and Migration

**DOI:** 10.3390/ijms242216285

**Published:** 2023-11-14

**Authors:** Paolo Zuccolini, Raffaella Barbieri, Francesca Sbrana, Cristiana Picco, Paola Gavazzo, Michael Pusch

**Affiliations:** Biophysics Institute, National Research Council, 16149 Genova, Italy; paolo.zuccolini@ibf.cnr.it (P.Z.); raffaella.barbieri@ibf.cnr.it (R.B.); francesca.sbrana@ibf.cnr.it (F.S.); cristiana.picco@ibf.cnr.it (C.P.); paola.gavazzo@ibf.cnr.it (P.G.)

**Keywords:** IK, KCa3.1, *KCNN4*, cancer, melanoma, pancreatic duct adenocarcinoma (PDAC), blockers, clotrimazole, senicapoc

## Abstract

Many studies highlighted the importance of the IK channel for the proliferation and the migration of different types of cancer cells, showing how IK blockers could slow down cancer growth. Based on these data, we wanted to characterize the effects of IK blockers on melanoma metastatic cells and to understand if such effects were exclusively IK-dependent. For this purpose, we employed two different blockers, namely clotrimazole and senicapoc, and two cell lines: metastatic melanoma WM266-4 and pancreatic cancer Panc-1, which is reported to have little or no IK expression. Clotrimazole and senicapoc induced a decrease in viability and the migration of both WM266-4 and Panc-1 cells irrespective of IK expression levels. Patch-clamp experiments on WM266-4 cells revealed Ca^2+^-dependent, IK-like, clotrimazole- and senicapoc-sensitive currents, which could not be detected in Panc-1 cells. Neither clotrimazole nor senicapoc altered the intracellular Ca^2+^ concentration. These results suggest that the effects of IK blockers on cancer cells are not strictly dependent on a robust presence of the channel in the plasma membrane, but they might be due to off-target effects on other cellular targets or to the blockade of IK channels localized in intracellular organelles.

## 1. Introduction

In recent years, ion channels have emerged as potential targets for cancer treatment [1,2,3,4]. This should not surprise, considering the multitude of physiological cellular processes in which they take part [5]. To present a few examples: changes in the membrane potential are important for the regulation of the cell cycle [6,7]; Cl^−^, K^+^ and Ca^2+^ channels are involved in apoptosis [8]; and the regulation of cell volume requires a class of specialized volume-sensitive channels [9,10]. Among the different ion channels that populate the membranes of cancer cells, many studies have focused on the Ca^2+^-gated K^+^ channel KCa3.1 (commonly known as IK), which is encoded by the gene *KCNN4* [11]. IK was first discovered in the 1950s by Gárdos, who observed that intracellular Ca^2+^ can enhance the K^+^ permeability of human erythrocytes [12]. The channel was later cloned and heterologously expressed in different cell types, allowing a detailed characterization of its biophysical properties and pharmacology [13,14,15]. IK opens in response to an increase in [Ca^2+^]_i_. The gating mechanism became clear with the cryo-EM structure solved by Lee and MacKinnon [16]. Briefly, IK is a homo-tetrameric protein displaying an architecture resembling that of non-swapped 6-TM K^+^ channels [16] with three characteristic cytosolic helices at the C-terminal end of S6 [16]. Pore opening is regulated by intracellular Ca^2+^ binding to calmodulin (CaM) [17], which is constitutively associated with the C-terminal cytosolic helices of the channel [16]. Most of the known IK blockers, including the antimycotic-derived ones, inhibit IK by directly binding the channel pore module just below the selectivity filter [16,18] (see Table 1). Such molecules have been proposed as potential cures for medical conditions in which the IK channel is directly involved.

It has been known since the late 1990s that the IK channel is blocked by the scorpion toxin charybdotoxin and by clotrimazole, which is a member of the imidazole antimycotics family [13,15,18,19,20]. Clotrimazole blocks the channel very efficiently but is also an inhibitor of cytochrome P450 (CYP) enzymes from different species (see for example [21,22,23,24,25]). In order to obtain a more selective molecule, Wulff and colleagues used a rational design strategy to develop a clotrimazole analog lacking the imidazole ring, which is strictly required for cytochrome P450 inhibition, obtaining the molecule known as TRAM-34 (IC_50_ = 20 nM) [26]. However, TRAM-34 displays a few shortcomings, which include a low oral bioavailability, even after enteric coating, and a short half-life [27,28]. Moreover, it has been reported that TRAM-34 can inhibit human and rat CYP isoforms although at relatively high concentrations [29]. Three years later, senicapoc, another IK inhibitor, was developed [30]. Senicapoc, when compared to TRAM-34, has a longer half-life, is more orally bioavailable in humans, has a lower IC_50_ (11 nM), displays an increased metabolic stability and no effects on CYP enzymes have been reported [18,27,30,31,32].

**Table 1:** Names, 2D structures and references of the compounds mentioned in the introduction.

Clotrimazole	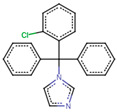	Reference [20]
Tram-34	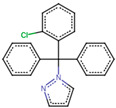	Reference [26]
Senicapoc	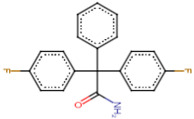	Reference [30]

As previously mentioned, IK expression has been reported to be altered in different types of cancer cells. Epigenetic dysregulation of the *KCNN4* gene, leading to a high-level expression of IK, has been correlated with the aggressiveness of lung cancer [33], and channel upregulation has been observed also in glioblastoma cells [34]. Moreover, channel expression and activity turned out to be important for the progression of the cell cycle, as observed for example in breast cancer and endometrial cancer cells [35,36]. It is indeed well known that potassium channels are involved in the regulation of the cell cycle of healthy and tumor cells [6]. In this scenario, different groups have employed IK blockers in the attempt to arrest the cell cycle of cancer cells and to reduce their migration. It was reported that 20 to 30 µM TRAM-34 can slow down the proliferation and the migration speed of lung cancer cells and that senicapoc, when administered in vivo, reduced the tumor mass in mice [33]. Experiments on intrahepatic cholangiocarcinoma cells showed that 40 µM TRAM-34 induced a reduction of ~50% on cell proliferation after 72 h and decreased invasiveness and migration; also in this case, senicapoc was able to reduce the tumor mass in vivo [37]. TRAM-34, at concentrations up to 40 µM, can arrest the cell cycle and therefore the growth of endometrial cancer cells; the same effect could be observed, at lower concentrations, with the less IK-specific clotrimazole [35]. The latter has been reported to block (at a concentration of 20 µM) breast cancer cells in the G1 phase [36] and to decrease the proliferation of pancreatic cancer cells [38,39]. Also in non-small cell lung cancer cells [40], cervical cancer cells [41], and triple-negative breast cancer cells [42], IK block reduced proliferation. It should be noted that the concentrations of the various IK blockers used in these cellular and in vivo studies are in the tens of µM range, which is much higher than the reported EC50 concentrations required to achieve channel block in patch-clamp experiments. The reason is that clotrimazole, TRAM-34 and senicapoc bind to serum proteins [31,40,43,44], such that their effective concentration is much lower in the presence of serum, which is commonly not present in the patch-clamp experiments.

In recent years, our lab has been studying ion channels expressed in different types of cancers [45,46,47]. In particular, we are interested in understanding the importance of these proteins in the viability, proliferation and migration of melanoma and pancreatic duct cancer cells.

In the present work, we characterized the impact of IK channel blockers on the proliferation and the migration of the metastatic melanoma cells WM266-4 [48]. A deeper knowledge of melanoma metastasis is indeed crucial for the battle against this type of cancer, as its outcome becomes worse when the tumor starts to invade other tissues [49]. The 5-year survival rate drops from 93% for stage IIIA to 32% for stage IIID [50], and for patients with stage IV metastatic melanoma, the median survival is less than one year [51].

Thus, understanding whether IK channel blockers act on cancer cells also through IK-independent mechanisms and whether a clear correlation between their effects and IK expression levels exists could help elucidate the molecular mechanisms underlying melanoma development. Channel modulators can affect cells in multiple ways. For example, we recently found that activators of the BK channel can change the intracellular Ca^2+^ concentration of melanoma cells and that the VRAC blocker DCPIB directly activates the BK channel as an off-target effect [46,52]. For this reason, in the present work, we also tried to address general questions regarding IK blockers action on cancer cells: are the obtained results solely due to channel blockade? Is there a correlation between the presence in the membrane of functional channels and the observed effects of IK blockers? To this purpose, we employed two different blockers and two different cell lines. We chose clotrimazole, known for having side effects, and senicapoc, one of the most IK-selective blockers available. The two compounds were tested in parallel on two cell lines: WM266-4 and the pancreatic duct adenocarcinoma (PDAC) line Panc-1, which is reported to express little or no IK protein and to lack IK-like currents [38,39,53] and therefore is regarded as a reference cell line. To our surprise, migration and proliferation were decreased in both cell lines regardless of IK expression levels. To obtain more insights into this unexpected phenomenon, we performed electrophysiology and calcium imaging experiments. The results suggest that indeed, the effect of IK blockers on cancer cells might not strictly depend on a robust presence of functional channels in the membrane.

## 2. Results

### 2.1. IK Blockers Reduce Viability of Both WM266-4 and Panc-1 Regardless of Expression Levels

**Figure 1:**
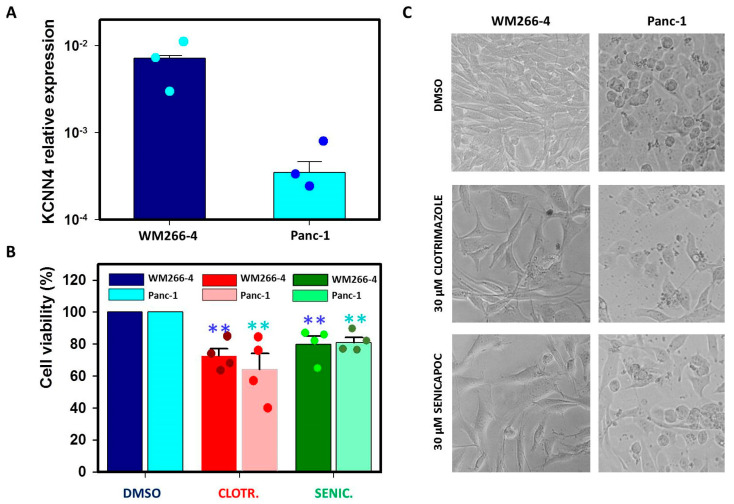
IK blockers affect WM266−4 and Panc−1 viability regardless of IK channel expression. (**A**) Relative *KCNN4* expression from RT-qPCR showing the difference in *KCNN4* mRNA levels between WM266-4 and Panc-1 (N = 3). (**B**) Results from MTT viability assays after 72 h of exposure to 30 µM clotrimazole, 30 µM senicapoc or the corresponding amount of DMSO (N = 4 for all). Data are reported as absorbance (570 nm) ratio drug-/DMSO-treated cells (color code reported in the legend). (**C**) Exemplary pictures from experiments in (**B**): WM266-4 and Panc-1 cells treated for 72 h with 30 µM clotrimazole, 30 µM senicapoc or the corresponding amount of DMSO. Significance level is indicated by two asterisks (*p* < 0.01).

In order to confirm the data reported in the literature regarding the low expression of IK in Panc-1 cells, we performed RT-qPCR experiments (Figure 1A). Such an analysis showed that the channel relative expression is much higher in WM266-4 cells compared to Panc-1, validating our idea of using these two cell lines to test for a correlation between IK blockers effects and channel expression. We therefore proceeded to test the impact of clotrimazole and senicapoc on cell viability using the MTT assays. Based on previous studies, cells were seeded in a multi-well plate and treated with 30 µM clotrimazole or 30 µM senicapoc or the corresponding volume of the solvent DMSO for 72 h. In the literature, it is reported that the concentration of serum in the medium can affect the impact of IK blockers on cancer cell proliferation. Indeed, it was shown that a decrease in the proliferation of PDAC cells could be obtained also at lower concentrations of IK inhibitors as long as the serum concentration was 1% [38]. We preferred to use higher concentrations of the compounds rather than modify such a critical factor for cell growth as the serum in the medium.

In agreement with its lack of specificity for IK, clotrimazole caused a reduction in viable cells with respect to the DMSO-treated controls in both WM266-4 and Panc-1 cells (Figure 1B,C; −27.4 ± 4.6% for WM266-4 and −36 ± 10% for Panc-1). To our big surprise, also the presumably IK-selective senicapoc induced a decrease in cell viability in both cell lines to a similar extent (Figure 1B,C; −20.2 ± 5.1% for WM266-4 and −19 ± 3.1% for Panc-1 with respect to DMSO-treated cells), in contrast with the results of channel expression analysis, which showed that the *KCNN4* mRNA levels are higher in WM266-4 compared to Panc-1.

### 2.2. Clotrimazole and Senicapoc Affect the Migration of WM266-4 and Panc-1

**Figure 2:**
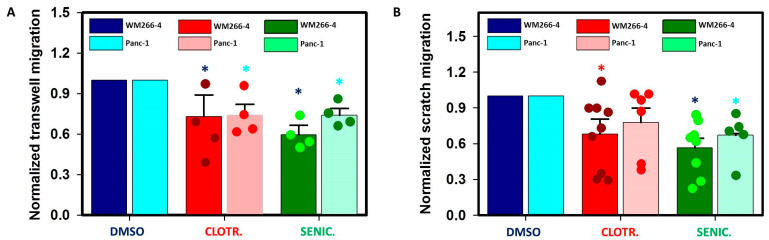
Clotrimazole and senicapoc decrease the migration of WM266-4 and Panc-1. (**A**) Migration rate from trans-well migration assays of cells exposed to 30 µM clotrimazole or 30 µM senicapoc with respect to DMSO-treated cells (N = 4 for all). Different cell lines and treatments are color coded as reported in the legend. (**B**) Relative increases of cell-covered areas at t = 24 h with respect to t = 0 from the same petri dish for WM266-4 (DMSO N = 9, clotrimazole N = 9, senicapoc N = 8) and Panc-1 (DMSO N = 6, clotrimazole N = 6, senicapoc N = 5). Data are significantly different from control for WM266-4, clotrimazole (*p* = 0.0128), WM266-4, senicapoc (*p* = 0.001), and Panc-1, senicapoc (*p* = 0.0246). Significance level is indicated by an asterisk (*p* < 0.05).

The MTT assay results suggested that IK channel expression is not proportional to the impact of clotrimazole and senicapoc on cell growth, so we went further by testing if such a correlation could be present regarding cell migration. To evaluate cell migration, we performed trans-well migration assays and wound (scratch)-healing assays on both WM266-4 and Panc-1, which is our reference for poor IK expression. To perform trans-well migration, cells were seeded on the upper side of the cell-permeable membranes (8 µm pores, see methods) in serum-free medium containing 30 µM clotrimazole or 30 µM senicapoc or the corresponding concentration of DMSO, while on the other side of the membrane, a complete medium served as a chemo-attractor to stimulate migration. Migrated cells were counted after 24 h (see Supplementary Figure S1A). In Figure 2A, we report the obtained data as the migration rate: the number of drug-exposed migrated cells compared to those which migrated after treatment with DMSO. The reduction in the migration induced by clotrimazole was similar between the two cell lines. For WM266-4, migrated cells were 73.9 ± 16% of those migrated in DMSO, while for Panc-1, they were 73 ± 8%. However, the IK-selective senicapoc impacted the migration of both cell lines as well: WM266-4 migrated cells were 59.4 ± 7.3% of those migrated in DMSO, while the ratio for Panc-1 was 74.2 ± 5%.

To further evaluate the impact of the employed compounds on WM266-4 and Panc-1 migration ability, we performed also scratch-healing assays. Cell migration from the scratch edges was monitored with a holographic microscope (see Supplementary Figure S1B). Figure 2B shows the movement of scratch edges as increases of cell-covered areas after 24 h long treatment with 30 µM clotrimazole or 30 µM senicapoc with respect to control DMSO-treated cells. In both cell lines, we observed a high variability in wound edges after 24 h long exposure to 30 µM clotrimazole. From experiments with senicapoc, we obtained more uniform datasets which showed that wound healing was significantly lower than that of DMSO-treated cells for both WM266-4 and Panc-1. Similarly to trans-well migration, WM226-4 cells were more affected by senicapoc than Panc-1 cells, which were nevertheless sensitive to the molecule. This indicated that the reduction in WM266-4 cell migration is possibly caused by a combination of IK blockage and an IK-independent effect, the latter being responsible for the Panc-1 migration decrease.

### 2.3. Characterization of WM266-4 and Panc-1 Whole-Cell Currents in High Intracellular Ca^2+^

**Figure 3:**
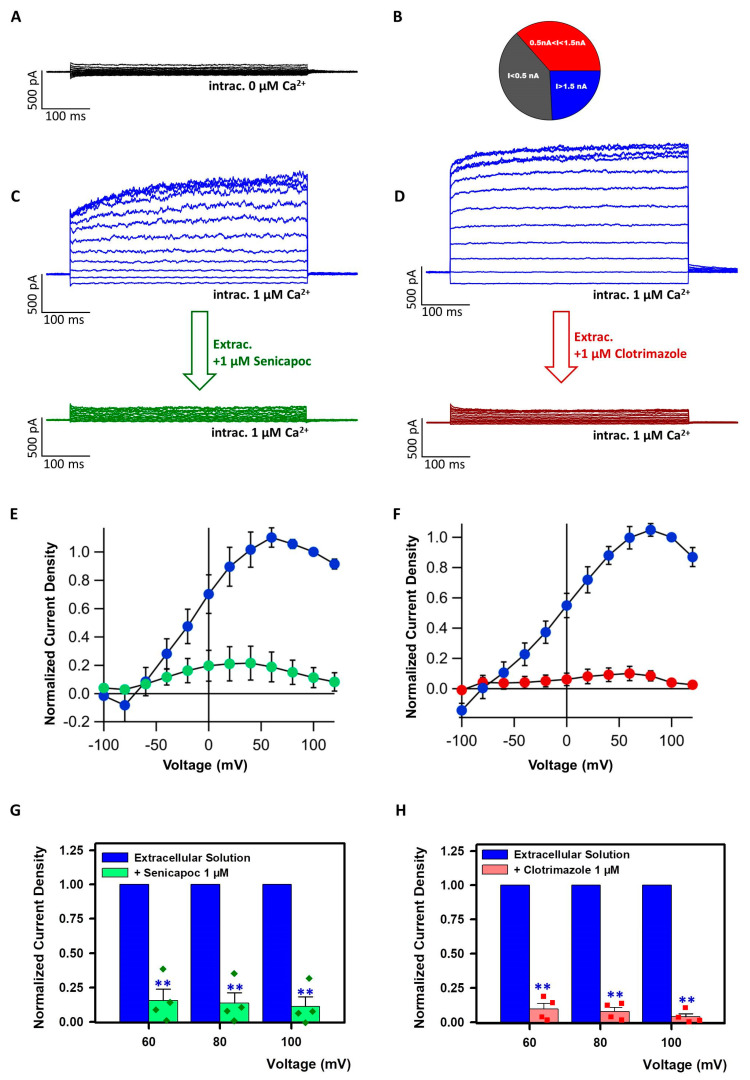
Ca^2+^−evoked whole-cell currents of WM266−4 cells. (**A**) Exemplary current traces of WM266-4 whole-cell currents when the patch pipette was filled with the Ca^2+^-free intracellular solution. (**B**) Distribution of current amplitudes in WM266-4 cells measured at 100 mV with 1 µM Ca^2+^ in the pipette solution. (**C**) Exemplary WM266-4 current traces from recordings with 1 µM Ca^2+^ in the intracellular solution; cells were perfused with standard bath solution (top) or with the same solution + 1 µM senicapoc (bottom). (**D**) Exemplary Panc-1 current traces from recordings with 1 µM Ca^2+^ in the intracellular solution; cells were perfused with standard bath solution (top) or with the same solution + 1µM clotrimazole (bottom). (**E**) Average normalized IVs of WM266-4 cells before (blue circles) and after (green circles) application of 1 µM senicapoc (N = 4 cells). Currents are normalized to the value at 100 mV. (**F**) Average normalized IVs of WM266-4 cells before (blue circles) and after (red circles) application of 1 µM clotrimazole (N = 4). Currents are normalized to the value at 100 mV. (**G**) Background-subtracted currents (background was calculated from the mean of 4 cells measured in Ca^2+^-free conditions) in the presence/absence of senicapoc normalized to the currents measured in standard bath solution at the same voltage in the same cells (mean ± SE, N = 4). (**H**) Background-subtracted currents in the presence/absence of clotrimazole normalized for the currents measured in standard bath solution at the same voltage in the same cells (mean ± SE, N = 4). Significance level is indicated by two asterisks (*p* < 0.01).

Our results indicated that the IK-selective compound senicapoc can impact the growth and the migration of WM266-4 cells, but also of Panc-1, in which IK is poorly expressed. Since the presence of mRNA coding for a given channel does not always correlate with the presence of the correspondent conductance in the membrane, we performed whole-cell patch-clamp experiments to assess the presence of Ca^2+^-activated currents with properties of IK channels in WM266-4 and Panc-1 cells. In WM266-4, we observed that 1 µM Ca^2+^ in the recording pipette triggered a voltage-independent current, which could not be detected when an intracellular solution with nominally 0 mM free Ca^2+^ was employed (Figure 3A–D). Currents reverted from negative to positive values at negative potentials as can be seen from the traces reported in Figure 3C,D, where pre- and post-pulse voltages correspond to the cell resting potential (around −50 mV). We observed a high variability in current amplitude among different cells, and some cells did not even display any Ca^2+^-activated current (out of 33 patched WM266-4 cells, 12 had currents at 100 mV smaller than 0.5 nA, 13 had currents between 0.5 and 1.5 nA, and 8 had currents larger than 1.5 nA; see Figure 3B). The Ca^2+^-elicited currents were strongly reduced by senicapoc (Figure 3C,E) and clotrimazole (Figure 3D,F) already at a concentration of 1 µM. Figure 3G,H report the degree of block by the two compounds of the Ca^2+^-evoked currents at the voltages corresponding to their maximal amplitude.

As previously mentioned, we observed a relatively large variability in the amplitude of the Ca^2+^-dependent currents, while the currents recorded in the absence of intracellular Ca^2+^ were small and more comparable among different cells. For this reason, the apparent degree of block is also variable between different cells, as background conductances influence the results more strongly in cells that express less IK current. To obtain a more accurate quantification of the inhibitory effect, we subtracted background currents from the currents recorded in the presence/absence of the blockers. Both senicapoc and clotrimazole strongly reduced the currents induced by 1 µM Ca^2+^. The features and the pharmacology of the above-described currents strongly suggested that they were carried by IK channels.

**Figure 4:**
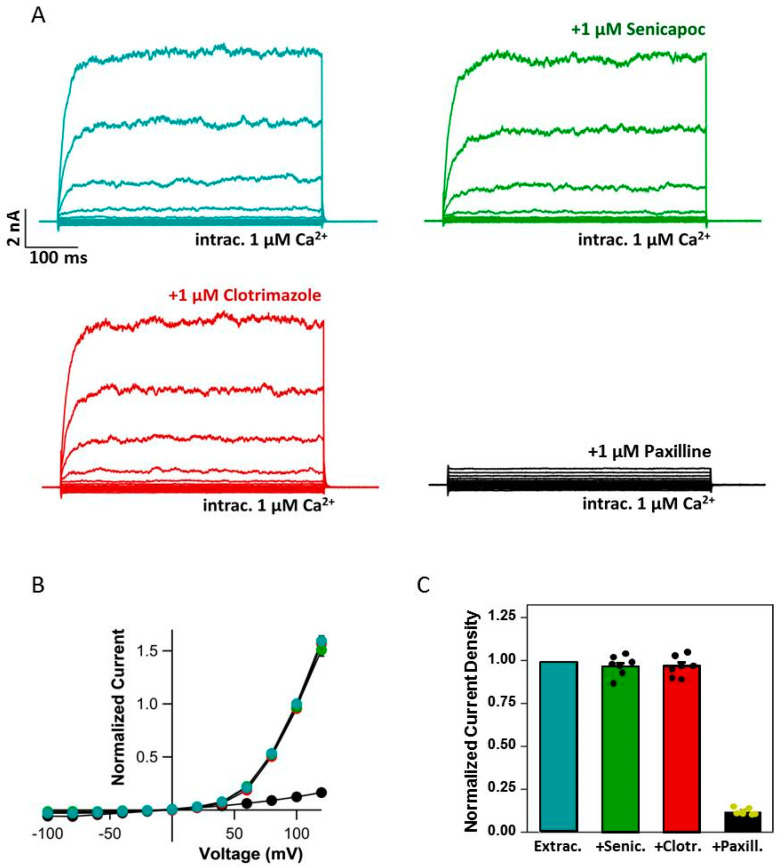
Panc−1 cells lack functional IK channels. (**A**) Current traces measured from a typical Panc-1 cell with 1 µM Ca^2+^ in the patch pipette in standard extracellular solution (top left), during perfusion with 1 µM senicapoc (top right), with 1 µM clotrimazole (bottom left) or 1 µM paxilline (bottom right). (**B**) Average normalized current voltage relationship in control conditions (turquoise symbols), in 1 µM senicapoc (green symbols, N = 7), 1 µM clotrimazole (red symbols, N = 7) and 1 µM paxilline (black symbols, N = 7) (currents are normalized to those measured in control conditions at 100 mV; error bars indicate SEM). (**C**) Average current density normalized to that measured in control conditions at 100 mV in the indicated conditions.

Since IK blockers had a significant impact also on viability and migration properties of Panc-1 cells, we needed to verify if these cells showed IK-like currents in the membrane as elsewhere reported [53]. We therefore applied 1 µM senicapoc (Figure 4A) or 1 µM clotrimazole (Figure 4B) to Panc-1 cells measured in the whole cell configuration with 1 µM intracellular Ca^2+^. Before drug application, large BK currents were seen in all patched cells [45,52], and none of the drugs had an appreciable effect (Figure 4A–C). Conversely, the subsequent application of 1 µM paxilline blocked most of the currents as expected (Figure 4A–C). Overall, these experiments exclude a significant presence of functional IK channels in Panc-1 cells.

### 2.4. IK Blockers Do Not Affect the Intracellular Ca^2+^ Concentration

**Figure 5:**
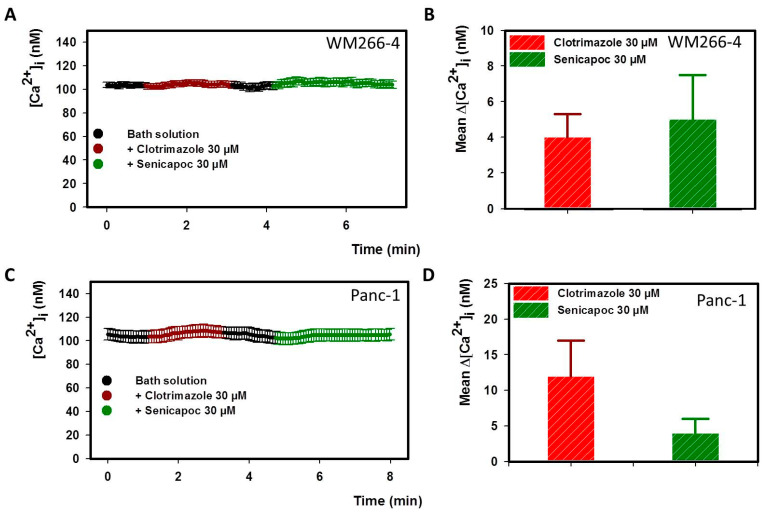
Senicapoc and clotrimazole do not alter the intracellular Ca^2+^ concentration. (**A**) [Ca^2+^]_i_ over time from 32 WM266-4 cells (color code in the legend). (**B**) Mean Δ[Ca^2+^]_i_ from (**A**) with respect to bath solution (color-coded as in (**A**), N = 32). (**C**) Mean [Ca^2+^]_i_ over time from 32 Panc-1 cells (color-coded as in (**A**)). (**D**) mean Δ[Ca^2+^]_i_ from (**C**) with respect to bath solution (color-coded as in (**C**), N = 32).

We have previously reported that K^+^ channel modulators can alter the intracellular calcium concentration ([Ca^2+^]_i_) of cancer cells [46]. It was also observed by other authors that the IK channel can regulate calcium entry: in prostate cancer cells, its activation induces calcium uptake through TRP channels by increasing the driving force for calcium [54]. To check if our results could be explained by [Ca^2+^]_i_ alterations, we performed Ca^2+^-imaging experiments on Panc-1 and WM266-4 cells testing whether clotrimazole or senicapoc can have some effects on [Ca^2+^]_i_. [Ca^2+^]_i_ was monitored over time with the fura-2 fluorescent probe before and after the application of 30 µM clotrimazole or 30 µM senicapoc. In Figure 5A,C the mean [Ca^2+^]_i_ values from these experiments are reported. Neither WM266-4 cells nor Panc-1 showed a significant variation of [Ca^2+^]_i_ after the application of the two molecules; rather, there were only small fluctuations that were never larger than 15 nM. The average Δ[Ca^2+^]_i_ values (with respect to the standard bath solution) after the perfusion of clotrimazole and senicapoc on WM266-4 cells were only 4 ± 1.3 nM and 5 ± 2.5 nM, respectively (Figure 5B, N = 32 cells). Such small variations in [Ca^2+^]_i_ were observed also for Panc-1: Δ[Ca^2+^]_i_ values were 12 ± 5 nM for clotrimazole and 5 ± 2 nM for senicapoc (Figure 5D, N = 32 cells). The lack of significant variations in [Ca^2+^]_i_ caused by the two compounds indicated that they induced neither the activation of a Ca^2+^ membrane conductance nor the release of Ca^2+^ from intracellular stores.

### 2.5. Clotrimazole and Senicapoc Do Not Alter the F-Actin Organization

**Figure 6:**
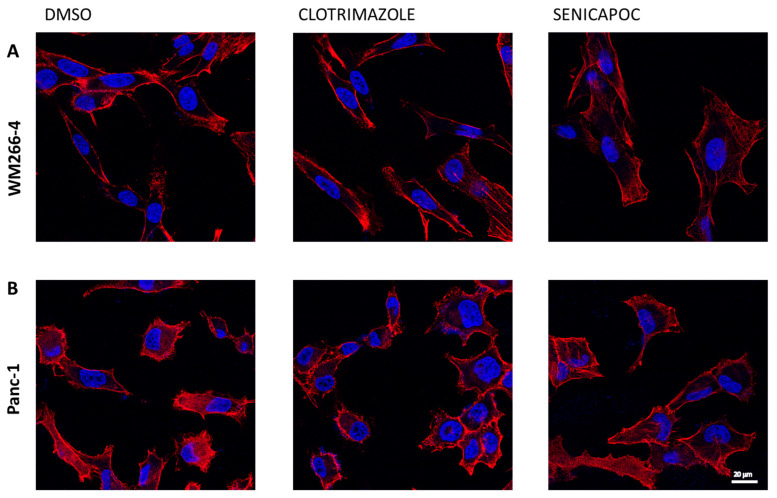
Effect of IK blockers on F-actin organization. WM266-4 (**A**) and Panc-1 (**B**) cells incubated for 24 h in vehicle alone or with 30 µM clotrimazole, 30 µM senicapoc or the corresponding volume of DMSO, which have subsequently been labeled with phalloidin (red) and DAPI (blue) and processed for fluorescence microscopy (see Section 4).

Another hypothesis we formulated to explain our unexpected results was that IK blocker treatments could affect cellular cytoskeleton organization. This idea emerged since the invasive migration of WM266-4 and Panc-1 was altered by the exposure to the tested molecules. Thus, in order to evaluate the effect of clotrimazole and senicapoc on Panc-1 and WM266-4 actin organization, we utilized phalloidin staining, which was able to bind the filamentous actin (F-actin). Phalloidin staining did not appear to modify both WM266-4 and Panc-1 cells after 24 h of treatment (Figure 6). However, changes in the F-actin organization could be detected after 72 h of treatment with 30 µM of clotrimazole or senicapoc (Supplementary Figure S2). These observations suggested that exposure to clotrimazole or senicapoc did not significantly alter the F-actin organization of Panc-1 and WM266-4 cells after 24 h.

### 2.6. Effect on Cell Viability of BA6b9, an Allosteric IK Blocker

**Figure 7:**
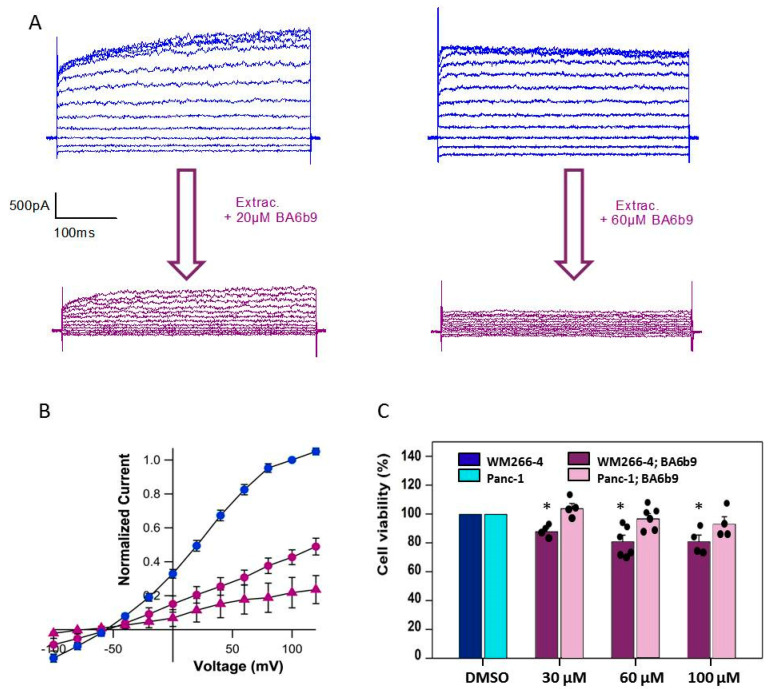
Effect of BA6b9 on IK−currents in WM266−4 cells and on viability in WM266−4 and Panc−1 cells. (**A**) Example currents measured from WM266-4 cells with 1 µM Ca^2+^ in the patch pipette in standard extracellular solution (top) and during perfusion with 20 µM (left) or 60 µM (right) BA69B. (**B**) Average normalized current voltage relationship in control conditions (blue symbols), in 20 µM BA6b9 (magenta circles), and 60 µM BA6b9 (magenta triangles) (currents are normalized to those measured in control conditions at 100 mV; N = 4 each, error bars indicate SEM). (**C**) Results from MTT viability assays after 72 h of exposure to DMSO (control) and the indicated concentrations of BA6b9 (N ≥ 4). Data are reported as absorbance (570 nm) ratio drug-/DMSO-treated cells (color-code reported in the legend. Significance level is indicated by an asterisk (*p* < 0.05).

Our data indicate that the clotrimazole-derived compound senicapoc exerts IK-independent effects on PDAC cells. Since clotrimazole and senicapoc share the same inhibition mechanism (they act on the channel pore), we decided to test if IK inhibitors with a different block mechanism were able to induce a decrease in Panc-1 viability similar to what we observed with clotrimazole and senicapoc. To this purpose, we employed an allosteric blocker called BA6b9, which acts on the CaM-PIP2-binding domain at the interface of the proximal carboxyl terminus and the linker S4–S5 [55]. We first tested whether the compound was able to inhibit IK currents in WM266-4 cells. As shown in Figure 7A,B, 20 µM BA6b9 inhibited around 60% of currents induced by 1 µM intracellular Ca^2+^, while 60 µM BA6b9 inhibited around 80%, with the residual currents being at least partially unspecific leak. The degree of inhibition is line what has been reported by Burg et al. [55]. We next repeated the viability assays using 30 µM, 60 µM and 100 µM BA6b9 employing the same experimental conditions as in Figure 1B. Interestingly, the compound did not induce any significant alteration in Panc-1 viability (Figure 7C), while we could only observe a slight but significant decrease in WM266-4 viable cells (Figure 7C).

## 3. Discussion

Like other K^+^ channels, IK was reported to have important roles in the proliferation and the migration of cancer cells; accordingly, clotrimazole, TRAM-34 and senicapoc were able to reduce cancer cells’ growth and migration ability as well as induce a decrease in tumor mass when administered in vivo [33,34,35,36,37,38,39]. It was also observed that the expression of IK varies throughout the cell cycle and seems to be important for its correct progression [36]. In these studies, the importance of IK for cancer cell tumorigenic processes has usually been highlighted by comparing aggressive IK-overexpressing cancer cells with blocker-exposed cells of the same line, siRNA IK-knocked-down cells or healthy cells from the same tissue of the tumor of interest.

Here, we chose a different but complementary approach to study the impact of IK blockers on the growth/migration of cancer cells, in particular on metastatic melanoma cells WM266-4. We selected clotrimazole and senicapoc as the two inhibitors: the former is already known to have side effects besides blocking the IK channel, while the latter is reported to be IK-selective. The idea of comparing the effects of these two compounds was to test if we could distinguish an IK-dependent from an IK-independent component in case the two added up. Moreover, we decided to employ Panc-1 cells from primary pancreatic cancer as a term of comparison, since they poorly express the IK channel. The latter idea turned out to be quite fruitful, as the most insightful results were obtained from the comparison of the two cell lines.

To estimate cell growth, we seeded the same number of Panc-1 and WM266-4 cells and performed MTT viability assays after a 72 h long treatment with clotrimazole, senicapoc or the corresponding amount of their solvent DMSO. The unspecific clotrimazole caused a decrease in viability in both WM266-4 and Panc-1 cells. This result is compatible with previous observations that clotrimazole inhibits the activity of cytochrome P450, a large family of heme-containing oxidases, which play essential roles in endogenous signaling and metabolic pathways. However, intriguingly, also senicapoc was able to induce a reduction in cell viability for both cell lines. This decrease was similar for WM266-4 and Panc-1, so it did not reflect the difference in IK expression assessed by RT-qPCR.

The ability to migrate through the 8 µM wide pores is a good way to estimate the ability of cancer cells to migrate and invade other tissues; therefore, we performed trans-well migration assays. We found that clotrimazole affected the migration ability of both cell lines, which was probably as a result of its lack of target specificity. Surprisingly, as observed for cell viability, also the IK-specific compound senicapoc affected both cell lines: the molecule induced a decrease in cell migration in both Panc-1 and WM266-4. Similar results were obtained with wound-healing assays. The latter were performed to observe the combination of the molecules’ actions on proliferation and migration at the same time, and for this reason, we did not employ proliferation inhibitors.

The data collected suggested that growth and migration might be hampered in WM266-4 cells via channel blockade by senicapoc but, in addition, also by an IK-independent effect visible in the control Panc-1. Therefore, our data seemed to diverge from the most accepted hypothesis about the action mechanism of IK blockers on cancer cells: that is, via inhibiting IK-mediated K^+^ conductances. We therefore performed patch-clamp experiments to evaluate the presence/absence of IK conductances in WM266-4/Panc-1 cells plasma membranes. In WM266-4 cells, we could measure Ca^2+^-triggered, voltage-independent currents whose features and pharmacology suggested that they were mediated by IK channels. The current density varied between different cells, which is compatible with the fact that IK over-expression in cancer cells is not constitutive but occurs only in certain phases of the cell cycle [35,36]. Upon exposure to senicapoc, the Ca^2+^-triggered currents dramatically dropped toward the level of the background, Ca^2+^-independent, ones. This suggested that IK was indeed the main mediator of the recorded currents. It excludes also the significant presence of other calcium-activated K channels, like for example BK, which is in accordance with what has been published earlier [53]. Currents like those recorded in WM266-4 could not be observed in Panc-1 cells when measured in the whole-cell configuration with Ca^2+^-enriched pipette solution. This confirms our hypothesis that the reduction in migration and proliferation observed in drug-exposed Panc-1 is not the direct result of IK blockade. It should, however, be kept in mind that the electrophysiological recordings only reveal IK channels localized in the plasma membrane. Thus, we cannot exclude putative effects on IK channels localized to intracellular membranes.

As outlined in the Introduction, the activation of IK can be expected to lead to an increase in [Ca^2+^]_i_ due to an increase in the electrochemical driving force. Conversely, the inhibition of IK might lead to a decrease in [Ca^2+^]_i_. However, in addition to these driving-force mediated effects, we know from our previous experience that K^+^ channel modulators can alter the intracellular calcium concentration of cancer cells in more unspecific ways [37,46,52]. For example, it could be that the two molecules could induce calcium uptake from the extracellular environment directly by opening Ca^2+^ channels. Another hypothesis was that the employed inhibitors were able to induce the release of Ca^2+^ from intracellular stores. In both cases, we would expect to see a large raise in [Ca^2+^]_i_ after acute exposure to clotrimazole and senicapoc. Calcium imaging experiments on WM266-4 and Panc-1 cells did not show any significant increase in [Ca^2+^]_I_ after the perfusion of the two compounds, excluding that they can have secondary effects similar to those observed, for example, for BK modulators [46].

Regarding the decrease in the trans-well motility of both WM266-4 and Panc-1, we reasoned that the compounds might affect the cytoskeleton organization of these cells. To test this hypothesis, cells were fixed and stained with phalloidin after exposure to clotrimazole or senicapoc. No changes in F-actin organization could be detected after 24 h treatment with senicapoc or clotrimazole, suggesting that blocker effects on migration are not correlated with large alterations of the F-actin organization. Thus, it is difficult to distinguish whether the rather marked effects on actin organization and cell size and shape seen after 72 h treatment are caused by a direct action on F-actin organization, are indirect consequences of other cellular alterations, or are simply linked with the loss of cell viability. Similar changes in F-actin organization have been reported for other ion channel modulators [56]; however, also in this case, it was difficult to distinguish between direct and indirect effects.

Taken together, the results we collected from all the experiments supported our hypothesis that clotrimazole and senicapoc can affect the carcinogenic behavior of PDAC cells independently of the presence of IK conductances in the plasma membrane. To test whether this is a specific shortcoming of clotrimazole-derived compounds, we tested the viability of WM266-4 and Panc-1 cells after 72 h of treatment with another molecule, namely BA6b9, which is an allosteric blocker of the IK channel. BA6b9, instead of binding the pore module of IK like antimycotic-derived compounds, hampers crucial interactions between S4–S5 linker, CaM and PIP2 [55]. We confirmed that BA6b9 inhibited IK currents in WM266-4 cells, necessitating however larger concentrations compared to clotrimazole and senicapoc, which is in agreement with the literature [55]. When we repeated MTT assays with BA6b9, we observed that Panc-1 cells were not affected by the exposure to this drug even at concentrations up to 100 µM. These experiments suggest that the reduction in viability observed in Panc-1 cells after treatment with senicapoc was due only to interactions of the molecule with secondary targets. Such secondary targets do not seem to be shared with BA6b9. Regarding WM266-4, we observed a slight viability decrease in BA6b9-exposed cells, but we believe that further studies will be required to determine if this drug can be used as a tool to reduce cancer cells viability and migration by targeting the IK channel. We used BA6b9 to test our hypothesis about the promiscuous behavior of senicapoc, but a deeper and systematic characterization of the impact of this compound on neoplastic cells can be an interesting topic for a whole new project.

The importance of IK channel expression for cancer progression has been suggested in a number of studies: knocking down the *KCNN4* gene can reduce carcinogenic behavior, and treatment with IK blockers had been reported to have an outcome comparable to that of knocking down the IK gene [37,38]. Therefore, we believe that IK blockade might reduce cancer cell growth. Nevertheless, in the present work, we discovered that relatively high concentrations of IK blockers can affect the proliferation and the migration also of cancer cells that do not display IK conductance in the plasma membrane. This suggests that IK blockade of plasma membrane-localized IK channels might not be the only mechanism by which senicapoc and other compounds exert their action on cancer cells. Another possible mechanism could be related to the presence of IK channels on the membrane of intracellular organelles like mitochondria [53], although this pathway is strongly dependent on the chemical nature of the drug. This hypothesis could be further analyzed in future studies. We believe that our findings should be taken into consideration when considering IK blockers (existing or to be developed in the future) as tools to slow down cancer growth and metastasis formation.

## 4. Materials and Methods

### 4.1. Cell Culture

Melanoma cell line WM266-4 (RRID:CVCL_2765) was cultured in MEM medium (Thermo Fisher Scientific, Waltham, MA, USA) supplemented with 10% FBS, 2 mM L-glutamine, 100 U/mL penicillin, 100 μg/mL streptomycin, and 1% non-essential amino acids (Sigma-Aldrich, St. Louis, MO, USA). The PDAC line Panc-1 (RRID:CVCL_0480) was grown in high-glucose DMEM (Thermo Fisher Scientific) enriched with 10% FBS, 100 U/mL penicillin, 100 μg/mL streptomycin, and 4 mM L-glutamine (Sigma-Aldrich). Cells were grown at 37 °C in a 5% CO2/95% air atmosphere (ESCO Lifesciences Group, Singapore). To split the cells into new flasks or to seed them in petri dishes and multi-well plates, they were washed with PBS (Euroclone, Pero, Italy) and then detached from the flask with 1 mL of trypsin–EDTA solution (Sigma-Aldrich).

### 4.2. RT-qPCR

Total RNA was extracted from WM266-4 or Panc-1 cells grown until sub-confluency in 25 cm^2^ flasks, using the PureLink RNA mini kit (Ambion Inc.-Austin, TX, USA), and 1 μg was reverse transcribed using the Super ScriptIV VILO cDNA synthesis kit (Thermo-Fisher Scientific, Milan, Italy). The obtained cDNAs were used as a template for RT-qPCR performed in the thermal cycler CFX Connect from Bio-Rad (Hercules, CA, USA). Gene expression was assessed by SYBR Green quantitative real time PCR using the SsoAdvanced Universal SYBR Green Supermix. The thermal protocol consisted of a denaturation step at 95 °C for 3 min, which was followed by 39 two-step cycles composed of a denaturation step at 95 °C for 10 sec and of annealing/extension at 55 °C for 30 sec. No template control (NTC) and no reverse-transcription control (NAC) were included to avoid false positives. Expression levels of the *KCNN4* target gene (encoding KCa3.1) were assessed in triplicate and then normalized to the expression of the housekeeping gene Actin. The following *KCNN4* primers were used: forward gctgcgtctctacctggtg; reverse cgatgctgcggtaggaag. Results were visualized with BIO-RAD software Bio-Rad CFX Manager-RRID:SCR_017251; Bio-Rad). We refer to the PCR cycle at which amplification fluorescence exceeds the background signal as the quantification cycle (Cq). Data are reported in the figures as relative expression with respect to the housekeeping gene GAPDH. This value is 2^−ΔCq^, where ΔCq is the difference between the Cq of the housekeeping gene and that of the target gene.

### 4.3. Materials

Clotrimazole, senicapoc and paxilline were purchased from Merck (Milan, Italy)and dissolved in DMSO to prepare stock solutions according to the information provided from the manufacturer. BA6b9 was kindly provided by Bernard Attali (Tel Aviv University). The compounds were added to solutions or mediums at the desired concentration prior to experiments. The final concentration of DMSO never exceeded 0.1%. MTT was purchased from Promega (Milan, Italy).

### 4.4. Cell Viability Assay (MTT)

WM266-4 and Panc-1 cells were seeded into 96-well plates (5 × 10^3^ cells per well). The following day, the wells medium was replaced with fresh medium containing the indicated amount of clotrimazole and senicapoc or the corresponding volume of DMSO. Incubation with IK blockers lasted 72 h, after which 20 μL of 3-(4,5-dimethylthiazol-2-yl)-2,5-diphenyltetrazolium bromide solution (MTT, Promega) was added to each well. Cells were incubated with MTT for 2 h at 37 °C. Relative cell viability was derived from the absorbance ratio (at 570 nm) between drug- and DMSO-exposed cells (plate reader from Molecular Devices, San Jose, CA, USA). For each experiment, each condition was tested in triplicate. Data are reported as cell viability normalized to the control condition (DMSO).

### 4.5. Trans-Well Migration Assay

WM266-4 and Panc-1 cells were suspended in serum-free medium containing the indicated final concentration of IK blockers or the corresponding volume of DMSO and then seeded into the upper side of cell culture inserts (8 × 10^3^ cells/insert) with cell-permeable membranes (8 µm pores, Sarstedt, Nümbrecht, Germany). The inserts were placed in a 24-well plate, in which the wells contained 600 µL of complete medium. In this way, the presence of serum in the medium below the membrane acts as a chemo-attractant. After 24 h, the cells were fixed with cold methanol and stained with crystal violet (Sigma-Aldrich). Unmigrated cells were scraped away from the upper layer of the membrane with a cotton swab. Pictures of the membrane bottom layers were taken with the help of a microscope (Nikon, Tokyo, Japan). Migrated cells were counted with the software FIJI [57] (version 1.53c, https://imagej.net/ij/, accessed on 15 August 2023). For each experiment, each condition was tested in duplicate. Data are reported as migrated cells with respect to the control condition (DMSO).

### 4.6. Scratch-Healing Assays

Panc-1 and WM266-4 cells were seeded in 35 mm petri dishes (2.5 × 10^5^ cells/petri). Then, 24 h after seeding, cells reached 100% confluency, and a wound in the cells layer was created with a 200 µL pipette tip. The cells’ medium was then removed and substituted with new medium enriched with 30 µM clotrimazole, 30 µM senicapoc, or the corresponding volume of DMSO. Cell migration was monitored in time under a holographic microscope Holomonitor M4 live cell imaging system (Phase Holographic Imaging, PHI AB, Lund, Sweden). Data analysis was performed using HStudio (PHI AB, Lund, Sweden). Pictures were taken after 24 h. Data are reported as increase in cell-covered areas after 24 h with respect to the control condition (DMSO).

### 4.7. Patch-Clamp Experiments

Cells were seeded in 35 mm dishes 24 h before the experiments. The standard intracellular pipette solution contained the following (in mM): 140 K-Asp, 4.3 CaCl_2_, 2.06 MgCl_2_, 5 EGTA, and 10 HEPES, and a pH of 7.2 was reached with KOH. The free calcium in this solution was calculated to be 1 µM using the Maxchelator program (Stanford University). Alternatively, a Ca^2+^-free pipette solution was used, in which CaCl_2_ was omitted and EGTA was increased to 10 mM. The extracellular solution contained the following (in mM): 150 Na-Asp, 5 KCl, 2 CaCl_2_, 1 MgCl_2_, 10 Glucose, and 10 HEPES, and a pH of 7.4 was reached with NaOH. The standard voltage-clamp protocol consisted of 500 ms long voltage steps, ranging from −100 to 120 mV in steps of 20 mV. The holding potential was set to the observed cell’s resting potential. To monitor the response of cells to different stimuli over time, we used a ‘time course protocol’, which administers to the cells a pulse of +100 mV lasting 50 ms. All currents were measured at 20 °C, using an Axon amplifier (Molecular Devices-San Jose, CA, USA) and filtered with a low-pass filter at 10 kHz. We used the acquisition software GePulse (freely available at http://users.ge.ibf.cnr.it/pusch/programs-mik.htm, accessed on 25 February 2022). Currents were digitized with a National Instruments DAQ interface (Austin, TX, USA). Current traces were further analyzed with the freely available Ana analysis program (http://users.ge.ibf.cnr.it/pusch/programs-mik.htm, accessed on 13 August 2021).

### 4.8. Calcium Imaging Experiments

Measurements of cytosolic calcium ([Ca^2+^]_i_) were performed using the fluorescent indicator fura-2 AM. Before the experiments, cells were incubated for 45 min at 37 °C with 5 μM fura-2 AM (Sigma-Aldrich) dissolved in the same extracellular solutions of patch-clamp experiments, adding 0.1% pluronic acid in order to improve dye uptake. Coverslips were then transferred on the stage of an inverted Nikon TE200 fluorescence microscope. Cells were excited at 340 and 380 nm at 0.5 Hz with a dual excitation fluorometric Ca^2+^ imaging system (Hamamatsu, Sunayama-Cho, Japan). Fluorescence emission was measured at 510 nm and was acquired with a digital CCD camera (Hamamatsu C4742-95-12ER). To monitor [Ca^2+^]_i_, the fluorescence ratio F340/F380 was used. Monochromator settings, chopper frequency, and data acquisition were controlled by a dedicated software (Aqua Cosmos/Ratio, version U7501-01, Hamamatsu, Japan). [Ca^2+^]_i_ was calculated according to Grynkiewicz et al. [58]. We used a dissociation constant value for the Ca^2+^/fura-2 complex of 140 nM.

### 4.9. Phalloidin Staining

Cells were treated with IK blockers for 72 h, washed with PBS and fixed in 4% formaldehyde for 15 min at RT. After permeabilization with 0.1% Triton X-100 in PBS for 5 min and washing, they were incubated in rhodamine–phalloidin solution (1:100 in PBS) and DAPI for 1 h and washed with PBS three times. Then, each sample was examined by confocal microscopy using a Leica STELLARIS 8 Falcon τSTED (Leica Microsystems, Mannheim, Germany) inverted confocal/STED microscope. The fluorescence image (1024 × 1024 × 16 bit) acquisition was performed using an HC PL APO CS oil immersion objective 100× (1.40 NA).

### 4.10. Data Analysis

Data are reported as mean ± SE. When bar charts are depicted, also individual data points are superimposed. When data are normalized, it is stated in the text or figure legends. Differences between data groups reported in the same graph were checked for statistical significance by means of a paired-sample *t*-test or ANOVA followed by Tukey tests for mean comparison (>2 groups, normal distribution). For statistical analysis, we used OriginLab (Northampton, MA, USA). Figures were prepared using Sigmaplot (Spss Inc.-Chicago, IL, USA). The chosen significance thresholds of 0.05 and 0.01 are indicated by an asterisk (*) and double asterisk (**), respectively. Cells to be patched, imaged or used for any purpose were chosen randomly.

## 5. Study Limitations

In the present study, no genetic knock-out or knock-down of the *KCNN4* gene has been performed, which would provide more direct evidence of *KCNN4* independent effects of KCa3.1 inhibitors on cancer cell viability and migration.

## Data Availability

The data presented in this study are available on request from the corresponding author.

## Supplementary Materials

The supporting information can be downloaded at https://www.mdpi.com/article/10.3390/ijms242216285/s1.
