# Supplementary material for: IK Channel-Independent Effects of Clotrimazole and Senicapoc on Cancer Cells Viability and Migration
Source: Int J Mol Sci. 2023 Nov 14;24(22):16285. doi: 10.3390/ijms242216285 (PMC10671816; doi:10.3390/ijms242216285)

Supplementary Material for

IK Channel-Independent Effects of Clotrimazole and Senicapoc on Cancer Cells Viability and Migration

Authors: Paolo Zuccolini, Raffaella Barbieri, Francesca Sbrana, Cristiana Picco, Paola Gavazzo and Michael Pusch

Affiliation: Biophysics Institute, National Research Council, 16149 Genova, Italy

Supplementary Figure S1. Example pictures of transwell (A) and wound-healing (B) assays. Typical pictures are shown for the indicated conditions. In B, yellow lines indicate the cell front at t=0, and green lines indicate the cell front at t=24 h.

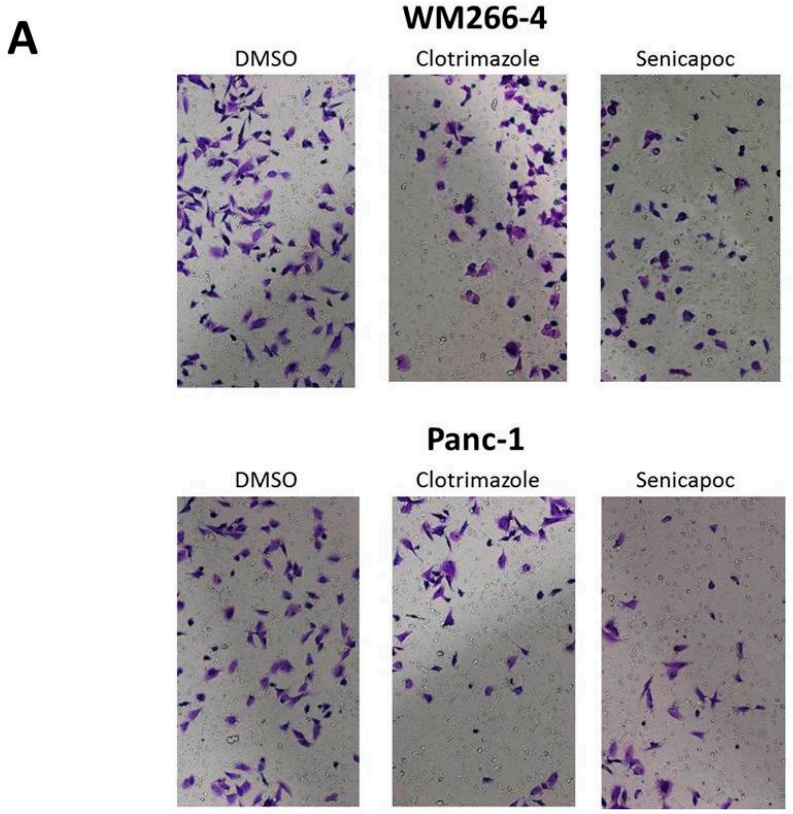

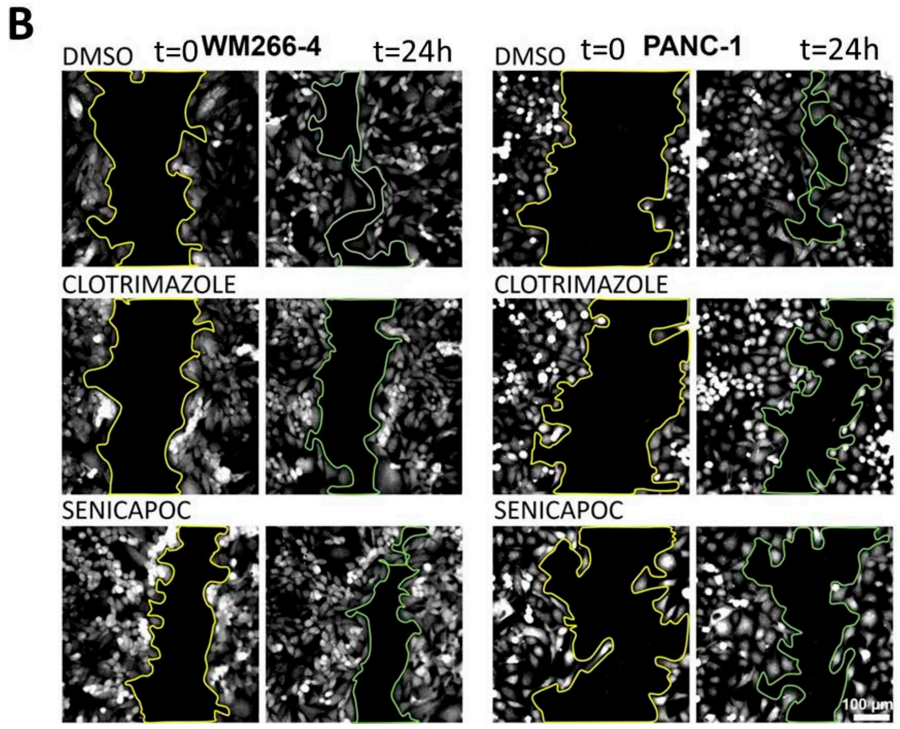

Supplementary Figure S2. Actin staining of WM266-4 and Panc-1 cells after 72 h treatment. Phalloidin staining after 72 hours of treatment with vehicle alone or with 30 µM of clotrimazole or senicapoc in WM266-4 and Panc-1 cells. In WM266-4 actin structure seems irregularly arranged and less organized. Treatments with IK blockers induced in Panc-1 cells a reduction of thin phalloidin labeled protrusions, resembling filopodia (see arrows in panel B) as well as an evident change in cell shape.

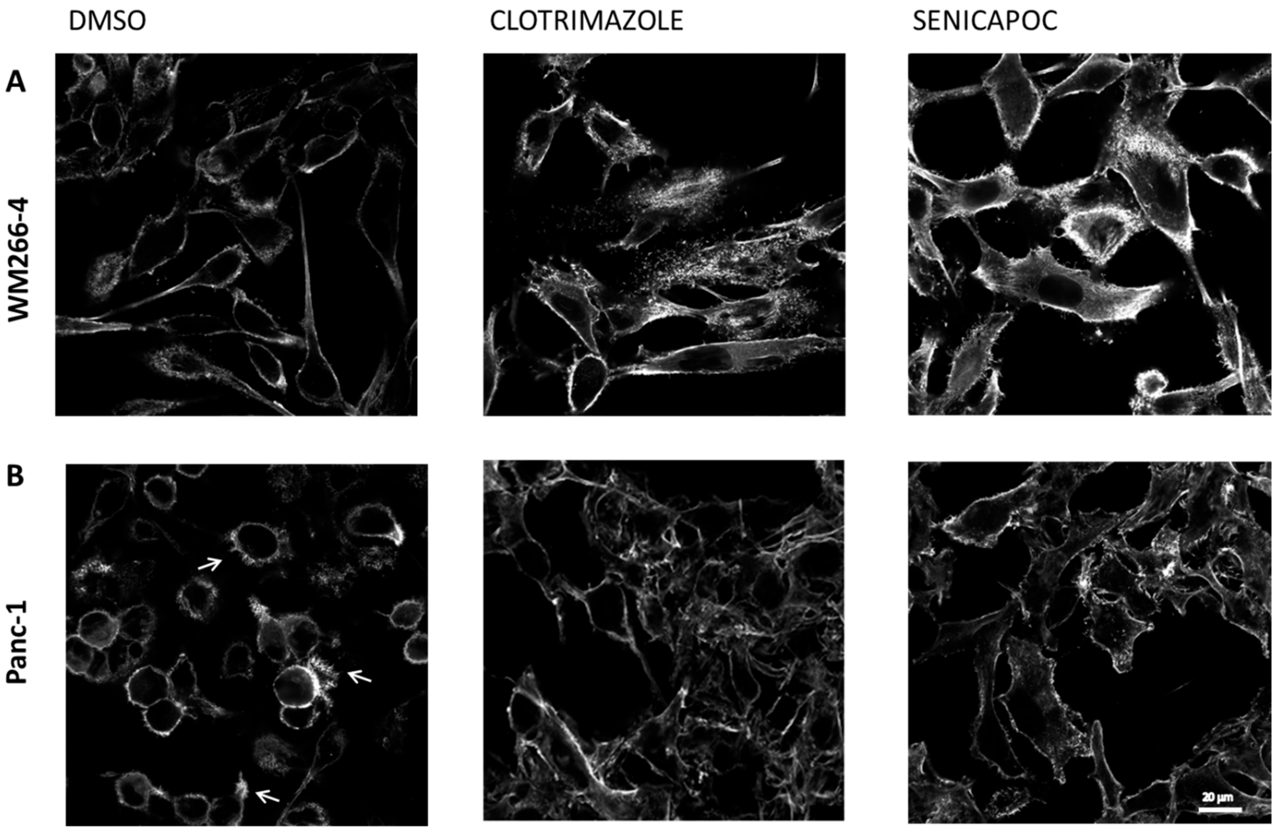

Supplement: Supplementary file 1 [file ijms-24-16285-s001.zip › ijms-2588531-supplementary.pdf]
